# Trends in Term-Equivalent Age Brain Volumes in Infants Born Across the Gestational Age Spectrum

**DOI:** 10.3390/children12081026

**Published:** 2025-08-04

**Authors:** Anouk S. Verschuur, Gerda van Wezel-Meijler, Selma Low, Ingrid M. Nijholt, Amy Metcalfe, Janice Skiffington, Donna M. Slater, Amy Bergeron, Elsa Fiedrich, Martijn F. Boomsma, Chantal M. W. Tax, Alexander Leemans, Lara M. Leijser

**Affiliations:** 1 Department of Radiology, Isala Hospital, Dokter van Heesweg 2, 8025 AB Zwolle, The Netherlands; 2 Department of Pediatrics, Section of Newborn Critical Care, University of Calgary, 3330 Hospital Drive NW, Calgary, AB T2N 4N1, Canada; 3 Image Sciences Institute, University Medical Center Utrecht, Heidelberglaan 100, 3584 CX Utrecht, The Netherlands; 4 Department of Neonatology, Isala Women and Children’s Hospital, Dokter van Heesweg 2, 8025 AB Zwolle, The Netherlands; 5 Department of Obstetrics and Gynecology, University of Calgary, 3330 Hospital Drive NW, Calgary, AB T2N 4N1, Canada; 6 Department of Physiology and Pharmacology, University of Calgary, 3330 Hospital Drive NW, Calgary, AB T2N 4N1, Canada; 7 Departments of Community Health Sciences and Medicine, University of Calgary, 3330 Hospital Drive NW, Calgary, AB T2N 4N1, Canada; 8 Division of Imaging and Oncology, University Medical Center Utrecht, Heidelberglaan 100, 3584 CX Utrecht, The Netherlands; 9 The Cardiff University Brain Research Imaging Centre, School of Physics and Astronomy, Cardiff University, Cardiff CF10 3AT, UK

**Keywords:** MRI, neonatal, brain, volume, gestational age, term-equivalent age

## Abstract

**Highlights:**

**Main findings**

• Lower gestational age is significantly related to larger cerebrospinal fluid volume.
• Trends of higher gestational age with larger volume of brain tissues are seen.

**Implication**

• Gestational age may drive early brain volumes with other neonatal clinical factors.

**Abstract:**

Purpose: Our understanding of the influence of preterm birth and related perinatal exposures on early brain development is limited, hampering personalized optimization of neuroprotective strategies. This study assesses the effect of gestational age (GA) at birth on brain volumes at term-equivalent age (TEA) in infants without overt brain injury born across the GA spectrum. Methods: A cohort of infants born across the GA spectrum (25–40 weeks’ gestation) underwent 3T brain MRI around TEA (40–46 weeks postmenstrual age). Eight brain regions, intracranial and total tissue volumes were segmented using MANTiS (morphologically adaptive neonatal tissue segmentation toolbox). Segmentations were visually quality-checked and excluded if segmentation failed. Absolute TEA volume in relation to GA was assessed using univariate and multivariate (correction for postmenstrual age) linear regression analysis. Statistical significance was set at *p* < 0.05. Post hoc scatter plots of brain volumes relative to intracranial volumes were created. Results: Fifty infants were included (mean GA = 35.0 [SD = 3.3, range = 25.7–40.1] weeks). A higher GA at birth was significantly related to lower cerebrospinal fluid (*p* = 0.004) and amygdala (*p* = 0.02) volumes; no significant relation was found between GA and other volumes. Post hoc analyses showed positive trends between GA and several brain structures, including total brain tissue, cortical gray matter, deep gray matter, hippocampus, cerebellum and brainstem volumes. Conclusions: Our results suggest that GA has an effect on TEA brain volumes that is independent of brain lesions, with lower GA being associated with smaller brain tissue volumes and significantly larger cerebrospinal fluid volume. Preterm birth and related exposures may thus affect early brain growth and contribute to neurodevelopmental challenges encountered by preterm-born children.

## 1. Introduction

Each year approximately 13 million infants are born prematurely (<37 weeks’ gestation) worldwide [1]. Many preterm infants face life-long challenges in neurodevelopment and social functioning, with the incidence and severity inversely related to gestational age (GA) at birth [2]. The causes of these challenges remain incompletely understood, especially in moderate–late preterm (MLPT; 32–36 weeks’ gestation) infants and infants without overt brain injury. Brain development is a complex and dynamic process with significant changes occurring throughout gestation. In the last trimester of pregnancy, the brain more than doubles in weight and triples in volume [3,4]. The developmental processes render the immature brain vulnerable to preterm birth and related perinatal complications and neonatal morbidity. The extrauterine environment exposes preterm-born infants to environmental stimuli, such as pain, noise, light and, to varying degrees, necessary interventions to manage complications, such as blood pressure fluctuations, infections, exposure to a pro-inflammatory environment due to stress and pain, nutritional challenges and respiratory support, sometimes with high-oxygen-level mechanical ventilation [5]. These exposures occur during a crucial time in brain development, potentially affecting brain growth and maturation [5].

The impact of the degree of prematurity at birth and with it the timing and intensity of adverse exposures on the vulnerable brain remain incompletely understood. Previous studies have predominantly examined the impact of very preterm birth (<32 weeks) on brain growth at term-equivalent age (TEA) as compared to full-term birth (>37 weeks), consistently reporting smaller brain volumes in very preterm infants, although some regional and methodological variability exists [6,7]. In contrast, relatively few studies have investigated brain volumes at TEA in MLPT infants (32–37 weeks), and the findings have been more inconsistent [8,9,10,11]. Given the smaller GA difference between MLPT and full-term infants, any volumetric differences may be more subtle. These subtle differences may potentially become more apparent when evaluating MLPT infants in view of both very preterm and full-term infants. To date, only two studies (utilizing data from the same prospective cohort) have assessed brain volumes in infants across the GA spectrum [10,12]. While larger cerebrospinal fluid (CSF) volumes have been consistently found at lower GA, relationships between GA and brain tissues are inconsistent or absent. For example, Alexander et al. reported significantly increasing white matter and brainstem volumes with the increase in GA, whereas Thompson et al. found no significant differences in brain volumes between neonatal sub-groups. These inconsistencies may be partly explained by the inclusion of infants with brain lesions, such as hemorrhagic and ischemic lesions, commonly seen in very preterm infants and shown to negatively affect brain volumes and neurodevelopmental outcomes in that population [7,13,14,15]. However, such moderate–severe brain lesions are less common in MLPT infants [16], and the causes of altered brain volumes and neurodevelopmental challenges in MLPT infants and very preterm infants without overt brain injury remain largely unclear.

We aimed to investigate the relationship between GA and brain volumes measured around TEA in a cohort of infants without visible brain lesions born across the GA spectrum (between 25 and 40 weeks). We hypothesized that a higher GA at birth is associated with larger tissue volumes and smaller CSF volumes at TEA.

## 2. Materials and Methods

### 2.1. Participants

Singleton preterm infants (<37^+0^ weeks) and full-term infants (39^+0^–40^+6^ weeks) with otherwise uncomplicated pregnancies and deliveries, born to families participating in the P3 Cohort (REB20-1635) and/or P3 High Risk Cohort (REB21-0712), were recruited into the P3 Brain Health Study (REB21-1446).

This study is an interim analysis of infants recruited between March 2022 and August 2024 who underwent brain MRI around TEA (40–46 weeks postmenstrual age [PMA]). Infants with genetic or congenital conditions were excluded. Full-term infants were excluded for additional reasons: pre-existing chronic condition or chronic steroid use in the mother, Neonatal Intensive Care Unit (NICU) admission or small size for GA (birthweight <3rd centile for GA). Baseline participant characteristics were collected. Written parental informed consent was obtained.

### 2.2. Data Acquisition

A research-dedicated 3 Tesla GE MR750W system was used for MRI acquisition. Toward the end of inclusion, the system (GE Ultra High Performance system) was upgraded. Scans after the upgrade were thoroughly checked, and calculated volumes were assessed using a scatter plot to limit the chance of bias in our analyses. Infants were scanned during natural sleep in the MedVac Vacuum Immobilization Bag. MiniMuffs (Natus, San Carlos, CA, USA) provided hearing protection. Infants with overt brain lesions, including (remnants of) intraventricular hemorrhage, post-hemorrhagic ventricular dilatation, periventricular hemorrhagic infarction, cerebellar hemorrhage, cystic white matter lesions, ≥6 punctate white matter lesions and arterial or venous infarction as visually assessed by LML and GvWM from conventional MRI (T1-, T2- and susceptibility-weighted imaging), were excluded prior to image analyses. T2-weighted images acquired with an axial fast spin echo sequence with a repetition time of 4400 ms, echo time of 120 ms, flip angle of 111, 320 × 320 acquisition matrix, 512 × 512 reconstruction matrix, pixel spacing of 0.375 × 0.375 and slice thickness of 2 mm (no gap) were used for volumetric analyses.

### 2.3. Brain Volumes

T2 MRI scans were visually quality-checked. Scans with motion and/or inhomogeneity artifacts and incomplete scans were excluded from analysis. T2 scans were skull-stripped using a brain extraction tool (BET, FSL version 6.0.3, FMRIB, Oxford, UK) with individually optimized thresholds (0.2 to 0.82) [17]. The BET results were visually checked by ASV, and extracerebral tissue was manually removed if still present.

**Figure 1:**
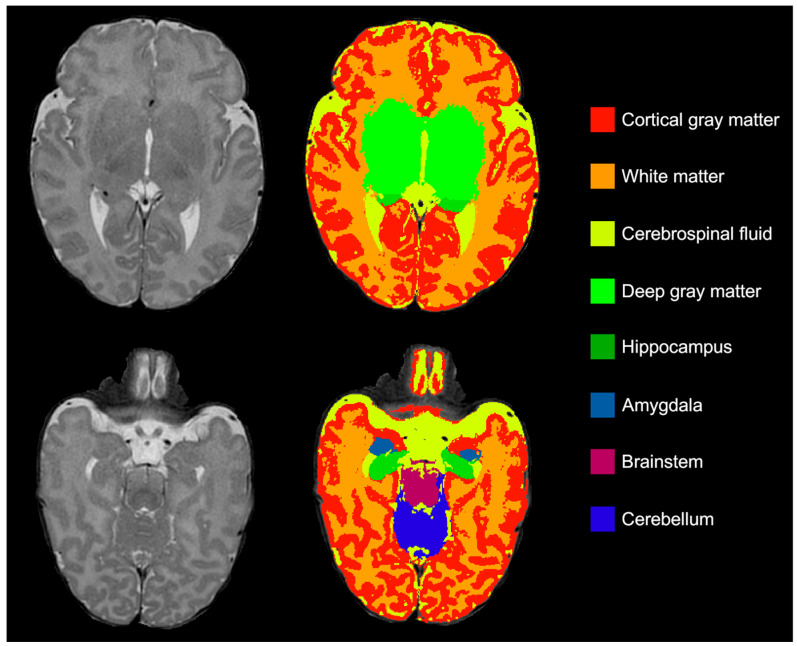
T2-weighed MRI (**left**) alongside color-coded segmented brain volumes (**right**).

Segmentation was performed using an adapted version of MANTiS (Murdoch Children’s Research Institute, Melbourne, Australia) [18], resulting in eight volumes that could be computed from the segmentations: the CSF, cortical gray matter, white matter, deep gray matter, hippocampus, amygdala, cerebellum and brainstem (Figure 1). Additionally, total tissue volume (excluding CSF) and intracranial volume (including CSF) were calculated by combining segmentation outputs. The segmentation results were visually checked by ASV. Mislabeling of brain structures was a reason to exclude the scan for further analyses.

### 2.4. Statistics

Prior to this study, a sample size calculation was performed using a pilot dataset of 20 scans. The relation between GA and brain volumes of all regions of interest was assessed using linear regression with correction for PMA at MRI. From the regression results, R-squared (*R*^2^) and R-squared change (∆*R*^2^) were extracted and used to calculate f-squared (*f*^2^), with $f^{2}=\frac{{∆R}^{2}}{1-R^{2}}$. The amygdala was the most conservative estimate with the smallest effect size (*f*^2^ = 0.23) and was used to estimate the largest sample size required to reach statistically meaningful results. G*power (version 3.1.9.7) was used to estimate the sample size using a *t*-test in linear multiple regression (single regression coefficient), with alpha 0.05, power 0.9, two predictors and effect size *f*^2^. This indicated a minimal sample size of 48 MRI scans across the GA spectrum to be required.

Baseline infant characteristics were calculated for included (preterm and full term) and excluded infants separately. Medians (with range) were calculated for all continuous variables to ensure consistency, but only GA and PMA were not normally distributed (assessed with the Shapiro–Wilk test). Frequencies (with percentages) were calculated for categorical variables. Characteristics of excluded and included infants were compared using chi-square tests (dichotomous variables) or the Mann–Whitney U-test (continuous variables).

Mean absolute volumes (in cm^3^) were calculated and presented with the standard deviation (SD) and range. To assess volumetric differences in infants born at different GAs, mean (SD) volumes for the following subgroups were calculated: extremely preterm (<28^+0^ weeks), very preterm (28^+0^–31^+6^ weeks), moderately preterm (32^+0^–33^+6^ weeks), late preterm (34^+0^–36^+6^ weeks) and full-term (39^+0^–40^+6^ weeks’ gestation).

Univariate and multivariate (with correction for PMA at MRI) linear regression analyses were performed to assess the relation between TEA brain volume and GA at birth. The results are displayed in a graph with B-values and 95% confidence intervals (95% CIs).

To further explore volumetric differences across GA, post hoc scatter plots of relative brain volumes were generated (including the trend line and 95% CI after bootstrapping 1000 times). The relative brain volumes were calculated by normalizing the brain volumes to the intracranial volume, partially accounting for increasing brain volumes with the increase in PMA.

Significance levels were set at *p* < 0.05, with a Bonferroni-corrected threshold of *p* < 0.005 to account for multiple testing. IBM SPSS statistics (version 28.0.1.1, IBM SPSS Statistics for Windows, IBM Corp., Armonk, NY, USA, Released 2017) was used.

## 3. Results

### 3.1. Infant Characteristics

**Figure 2:**
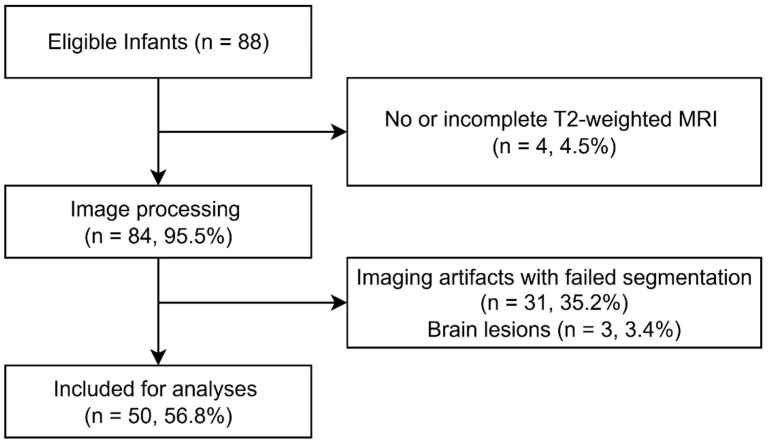
Inclusion flowchart indicating number of eligible infants, reasons for exclusion and final sample size. Percentages refer to the proportion that the respective number of infants represents of eligible infants.

For this interim analysis, 88 infants were eligible. After data quality assessment and image processing, the scans of four infants (4.5%) were excluded due to missing or incomplete T2-weighted MRI and 31 infants (35.2%) due to severe segmentation errors caused by motion artifacts, noise or scan inhomogeneity (Figure 2). Three further infants (3.4%) were excluded because of brain lesions (intraventricular hemorrhage with post-hemorrhagic ventricular dilatation, cerebellar hemorrhage and multiple connatal cysts). A total of 50 infants were included for analyses. The need for admission to a neonatal unit after birth and intensity of care required (i.e., intensive care versus high care) was significantly different between included and excluded infants (*p* = 0.01, Table 1), with more excluded infants admitted to a neonatal intensive care unit. No unanticipated differences in participant characteristics were found between preterm and full-term infants (Table 1).

### 3.2. Analyses

**Figure 3:**
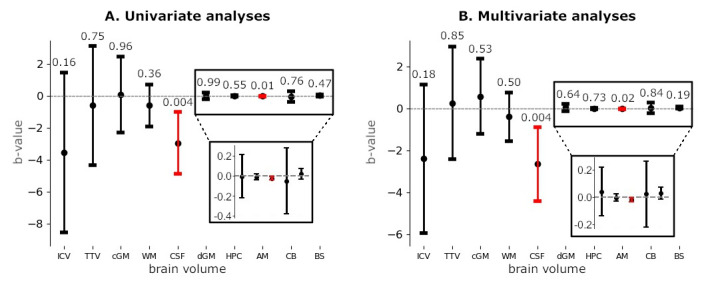
Results of univariate (unadjusted) linear regression (panel (**A**)) and multivariate (adjusted) linear regression (panel (**B**)), analyzing relation between gestational age at birth and brain volume measurements around TEA. The multivariate analysis is corrected for postmenstrual age at MRI. Regression coefficients, represented as B-values, above 0 reflect increasing volumes with the increase in GA and below 0 reflect decreasing volumes with the increase in GA. *p*-values are provided above the 95% confidence interval bars. Significant results are visualized in red. The boxes provide an enlarged view of the five smaller brain structures. ICV: intracranial volume; TTV: total tissue volume; cGM: cortical gray matter; WM: white matter; CSF: cerebrospinal fluid; dGM: deep gray matter; HPC: hippocampus; AM: amygdala; CB: cerebellum; BS: brainstem.

Mean (SD) volumes for included infants and per GA subgroup are presented in Table 2. Linear regression analyses revealed that CSF and amygdala volumes significantly decreased with the increase in GA in both the univariate and multivariate analyses (Figure 3A and Figure 3B). After Bonferroni correction, differences in amygdala volume were no longer statistically significant. No significant relationships were found for the other volumes.

**Figure 4:**
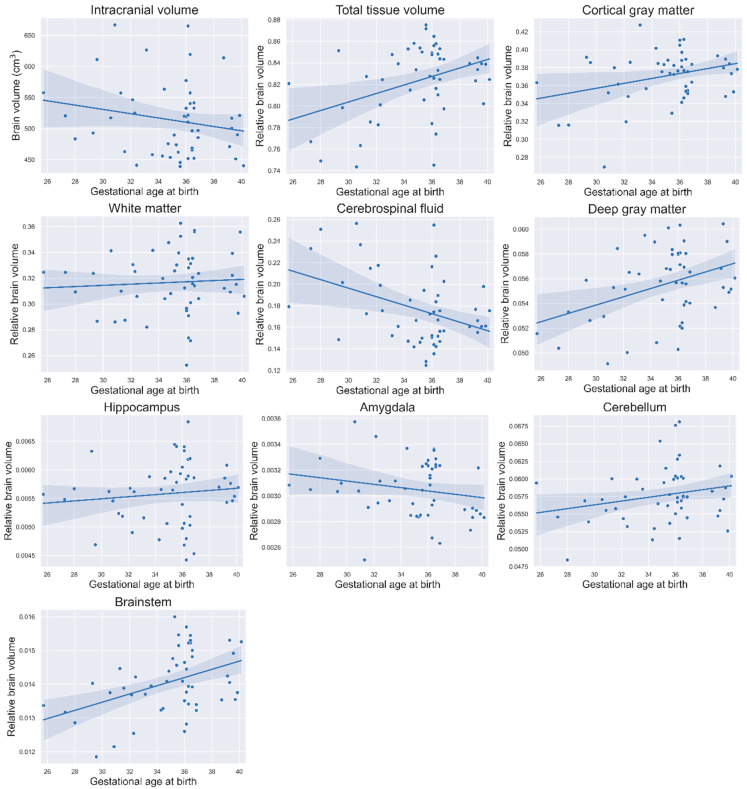
Post hoc scatter plots of relative brain volumes in relation to gestational age. Blue surface areas show the 95% confidence interval.

In post hoc plots, trends were observed for most relative volumes (Figure 4). Total tissue volume, cortical gray matter, deep gray matter, hippocampus, cerebellum and brainstem volumes increased with the increase in GA, while intracranial volume (absolute volumes), CSF and amygdala volumes decreased with the increase in GA. No effect of GA was seen for white matter volumes. Additionally, the scatter plots showed a considerable variation in brain volumes among infants born between 34 and 37 weeks’ gestation, compared to more uniform volumes in infants born >37 weeks’ gestation (Figure 4).

## 4. Discussion

To our knowledge, our study is the first to assess the relationship between GA and early brain growth in a cohort of infants born across the GA spectrum without visible brain lesions. While we observed significantly decreased CSF and amygdala (before Bonferroni correction) volumes at TEA with the increase in GA at birth, we found no relation for the other brain structures. Post hoc plots revealed trends of increasing and decreasing volumes with the increase in GA, suggesting that GA has an effect on TEA brain volumes independent of brain lesions.

Our results align with the findings of Thompson et al., who only found significantly larger TEA CSF volumes in the preterm sub-groups compared to full-term groups and Alexander et al., who observed decreasing CSF volumes with the increase in GA [12]. While Alexander et al. additionally identified statistically significant regional gray and white matter changes with GA, they did not find associations with total cortical gray matter, intracranial or total tissue volume [12]. Their inclusion of infants with brain lesions, potentially independently affecting brain volumes, and distinction between hemispheres may explain their more pronounced findings [12].

The negative association between GA and CSF volume at TEA observed in our study is consistent with previous neonatal MRI studies, which have reported increased CSF or ventricular volumes in infants born earlier in gestation [9,10,14,19,20]. This pattern is often interpreted as a delay or disruption in brain growth and maturation, leading to enlargements of CSF spaces in view of the continuing growth of the skull. Our multivariate analyses suggest that the (non-significant) trend toward decreasing intracranial volume with the increase in GA is primarily driven by changes in CSF volume. Brain tissue volumes showed only minor deviations from zero, indicating that the increase in CSF volume does not correspond to a substantial reduction in brain tissue volume. Similar findings were previously reported by Boardman et al. [19], who observed that preterm birth was associated with increased CSF spaces independent of tissue volume loss. Interestingly, amygdala volumes also significantly decreased with the increase in GA. This may reflect the amygdala’s sensitivity to heightened sensorimotor influences, and potential noxious stimuli preterm infants are exposed to earlier and for longer duration until MRI at TEA than full-term infants [21,22,23]. For example, Mueller et al. found that larger amygdala volumes in very preterm infants were associated with differences in social functioning at age five and that neonatal procedural pain moderated these associations [23]. However, findings in this current study should be interpreted with caution. After Bonferroni correction, the amygdala volume differences did not remain significant. Moreover, the amygdala’s small size and the challenges associated with segmentation may also have introduced a measurement error [18].

Post hoc plots revealed trends of increasing cortical and deep gray matter and cerebellar volumes with the increase in GA, while white matter volumes appeared unaffected. This contrast may be explained by the timing of brain developmental processes in relation to preterm birth: neurogenesis is largely complete around 28 weeks’ gestation, followed by processes such as synaptic pruning and apoptosis—processes that primarily shape gray matter by refining neural circuits and regulating neuronal populations [24]. Consequently, gray matter is particularly vulnerable to dysmaturation and impaired growth in preterm infants, even in the absence of brain lesions or lesions in other cerebral tissues [25,26,27]. Furthermore, the cerebellum undergoes rapid development, with mean volumes increasing 4-fold, between 28 and 36 weeks’ gestation [28]. This rapid growth makes the cerebellum highly vulnerable to injury; for example, even small amounts of blood from supratentorial intraventricular hemorrhage seeping into the subarachnoid spaces can impair cerebellar growth and maturation [29]. These dynamic and complex processes heighten the brain’s vulnerability to preterm extra uterine exposure, often accompanied by perinatal complications, neonatal morbidity and interventions at a vulnerable time.

Despite observing trends between GA and early brain volumes in post hoc analyses, the primary analyses did not yield statistically significant results. This suggests that GA may not be the main determinant of brain growth around TEA, but that volume differences in other studies are largely related to brain injury and subsequent interventions. Padilla et al. [30] found that extremely preterm infants without focal brain lesions had smaller gray matter volumes at TEA compared to full-term infants but also reported significant variations with the presence of other perinatal risk factors [30]. The findings from this study also suggest that GA may not be the main determinant of brain growth around TEA.

There is a great deal of heterogeneity within the preterm population concerning illness severity, required interventions and perinatal and genetic factors; all of which likely contribute to variability in brain development. Each infant’s unique medical and environmental circumstances and genetic predisposal may exert a greater influence on TEA brain volumes and/or neurodevelopmental outcome than GA alone [31]. Bouyssi-Kobar et al. [32] compared brain volumes between a preterm and fetal cohort and found decreased volumes in preterm infants compared to fetuses of the same gestation, highlighting the potential influence of clinical risk factors [32]. In addition, while small volumetric differences around TEA may not be statistically significant, they may predispose to significant deviations in childhood or adolescence [33] and potentially influence neurodevelopmental outcomes [34]. The small volume differences we observed may indicate that multiple brain developmental processes are falling behind, potentially sensitizing the brain to subsequent adverse exposures or causing the initial disruption to have a prolonged effect. Furthermore, the considerable variation in relative brain volumes among MLPT infants compared to the more uniform volumes observed in full-term infants stands out (Figure 4). This variability may be explained by the different degrees of illness in MLPT infants. While most do well after birth, some experience a more difficult start due to complications. These results mean that we may need to focus on sub-optimal brain development and optimization of interventions to inform novel or improved neuroprotective strategies, e.g., reducing painful procedures, reducing stressful exposures, increasing neuroprotective care (kangaroo care and minimal handling) and improving socioeconomic status.

Several limitations of this study should be acknowledged. First, this interim analysis was based on a relatively small sample size. While pilot data supported sufficient statistical power for the primary analyses, the limited cohort size restricted our ability to explore potentially important covariates such as sex, birth weight, head circumference, mode of delivery, underlying causes of prematurity, parental socioeconomic status, neonatal nutrition and early medical complications. Incorporating additional covariates into our multivariate model poses a risk of overfitting. Second, the sample was skewed toward MLPT infants, with very and extremely preterm infants underrepresented. While this selection likely reduced confounding by limiting the impact of severe neonatal illness and medical interventions, it also narrowed the generalizability of our findings. The cohort, although representative of the broader preterm population in which MLPT infants dominate (80%), may not fully reflect the spectrum of brain development in preterm infants with more complex clinical courses. Infants excluded from the analysis for limited T2 image or segmentation quality were more frequently admitted to higher level neonatal units (including NICUs), a difference primarily driven by the greater proportion of very and extremely preterm infants requiring intensive care. This was also seen with excluded infants being more frequently admitted to higher level neonatal units (including NICUs) and with the majority being born very or extremely preterm. The uneven GA distribution may also have affected the sensitivity to detect subtle differences across GA. Third, although beyond our control, the MRI scanner was upgraded midway through our study. Unfortunately, a formal statistical analysis comparing paired volumetric measurements from individual infants before and after the upgrade was not feasible. Instead, a visual quality check and scatter plots confirmed that the brain volumes were consistent before and after the upgrade. Finally, it is important to emphasize that while methodological choices, such as strict inclusion criteria and focused analyses, were made to ensure data quality and interpretability, larger and more harmonized studies will be essential to draw definitive conclusions. Reducing variability in methodological practices across studies and consideration of potential clinical covariates will be essential for gaining a clearer understanding of the nuanced effects of GA and related factors on early brain development.

More broadly, in future research, it is important to explore both quantitative and qualitative measures of brain maturation in the preterm population. For example, integrating multiple assessment methods, including clinical evaluations, brain volumes and advanced imaging techniques such as diffusion MRI, functional MRI and radiomics analysis, could provide a more comprehensive understanding of the impact of prematurity on brain development. Shifting focus from volumetric metrics to the quality of brain maturation, such as microstructural development and connectivity, may provide more meaningful insights into early developmental processes. For example, structural and functional MRI techniques to assess brain connectivity and functional maturation could offer a deeper insight into the complex developmental trajectories associated with prematurity. A better understanding of these processes could help improve early identification of infants at risk for adverse neurodevelopmental outcomes and inform more targeted, personalized interventions.

## 5. Conclusions

Significant decreases in CSF and amygdala volumes were observed in relation to GA, while for several other brain regions trends to both increasing and decreasing brain volumes with the increase in GA were seen. These findings may suggest that preterm birth affects brain growth independently from overt brain injury. These findings emphasize the importance of further investigating the impact of clinical and early neurodevelopmental factors on brain volumes, qualitative brain measures and neurodevelopmental outcomes to ultimately inform novel and improved neuroprotective strategies. Larger, harmonized studies that integrate relevant clinical covariates and employ both quantitative and qualitative neuroimaging methods will be critical to advancing our understanding of the complex neurodevelopmental challenges preterm infants face.

## Figures and Tables

**Table 1:** Participant characteristics compared between included and excluded infants and between preterm and healthy term born infants.

	Included Infants	Excluded Infants	*p*-Value	Preterm Infants	Term Born Infants	*p*-Value
	n = 50	n = 38		n = 42	n = 8	
**Neonatal period**						
Gestational age in weeks, median (range)	36.0 (25.7–40.1)	36.1 (26.3–40.3)	0.923	35.5 (25.7–36.9)	39.4 (38.7–40.1)	**<0.001**
Birth weight in grams, median (range)	2315 (915–4280)	2388 (900–4060)	0.723	2133 (915–4280)	3346 (2880–3970)	**<0.001**
Birth weight percentile, median (range)	45.0 (1.0–94.0)	52.5 (4.0–96.0)	0.242	45 (1.0–94.0)	47.0 (16.0–91.0)	0.990
Small for GA <10th percentile, n (%)	6 (12)	2 (5.3)	0.276	6 (14.3)	0 (0)	0.254
Small for GA <3rd percentile, n (%)	1 (2)	0 (0)	0.381	1 (2.4)	0 (0)	0.659
z-scores of body weight	0.02 (−2.35–3.26)	0.065 (−1.74–1.81)	0.539	0.075 (−2.35–3.26)	−0.075 (−0.99–1.33)	0.708
Head circumference in cm, median (range)	31.8(24.5–37.0) ^a^	31.7 (24.5–37.0) ^b^	0.470	31.5 (24.5–37.0) ^c^	34.5 (34.0–35.0)	**<0.001**
Sex			0.119			0.368
Male, n (%)	32 (64)	18 (47)		28 (67)	4 (50)	
Female, n (%)	18 (36)	20 (53)		14 (33)	4 (50)	
Admission level			**0.01**			**<0.001**
No admission, n (%)	17 (34)	18 (47)		9 (21)	8 (100)	
Admission to high care, n (%)	23 (46)	6 (16)		23 (55)	0 (0)	
Admission to intensive care, n (%)	10 (20)	14 (37)		10 (24)	0 (0)	
**Characteristics at MRI**						
PMA at MRI in weeks, median (range)	42.2 (40.0–46.9)	42.1 (40.0–46.9)	0.730	42.0 (40.0–46.9)	42.4 (41.0–45.0)	0.649
PNA at MRI in weeks, median (range)	6.9 (1.4–20.7)	6.1 (1.4–19.1)	0.846	7.4 (3.4–20.7)	3.1 (1.4–6.3)	**<0.001**
Weight in grams, median (range)	3775 (2210–5330)	3850 (2690–5470)	0.601	3708 (2210–5330)	3985 (3060–4240)	0.507
Head circumference in cm, median (range)	36.0 (33.5–39.5) ^a^	36.5 (34.5–40.5) ^b^	0.810	36.0 (33.5–39.5) ^d^	36.3 (35.0–37.5) ^e^	0.606

n: number; SD: standard deviation; ^a^ values for n = 48; ^b^ values for n = 37; ^c^ values for n = 40; ^d^ values for n = 41, ^e^ values for n = 7; TEA: term-equivalent age; PMA: postmenstrual age; PNA: postnatal age. Bolded values are significant.

**Table 2:** Mean (SD) and range of measured brain volumes by GA group.

	Mean Volume (SD), in cm^3^	Min–Max, in cm^3^	EP (n = 2), Volume (SD) in cm^3^	VP (n = 7), Volume (SD) in cm^3^	MP (n = 5), Volume (SD) in cm^3^	LP (n = 28), Volume (SD) in cm^3^	FT (n = 8), Volume (SD) in cm^3^
ICV	513.81 (58.31)	439.26–666.92	539.22 (26.25)	544.59 (75.59)	519.48 (74.39)	507.16 (53.78)	500.24 (54.22)
TTV	422.22 (42.89)	362.13–531.22	428.62 (41.38)	425.67 (59.42)	425.53 (64.61)	421.97 (35.38)	416.41 (48.17)
cGM	190.55 (27.16)	139.32–267.88	183.51 (27.03)	193.85 (41.61)	191.77 (43.12)	190.19 (22.44)	189.92 (23.29)
WM	162.01 (15.19)	133.04–193.36	175.03 (8.51)	161.51 (20.30)	163.92 (18.61)	161.76 (11.90)	158.85 (21.08)
CSF	91.59 (24.46)	55.60–169.54	110.61 (15.13)	118.92 (26.55)	93.95 (18.83)	85.19 (23.74)	83.83 (9.35)
dGM	28.43 (2.51)	24.64–35.33	27.50 (1.79)	28.82 (2.67)	28.79 (4.13)	28.48 (2.34)	27.93 (2.42)
HPC	2.86 (0.33)	2.18–3.64	2.98 (0.18)	2.94 (0.38)	2.82 (0.33)	2.84 (0.36)	2.83 (0.30)
AM	1.57 (0.21)	1.25–2.10	1.65 (0.09)	1.72 (0.25)	1.62 (0.24)	1.55 (0.19)	1.43 (0.18)
CB	29.61 (3.80)	23.40–38.14	30.78 (3.35)	29.79 (4.57)	29.55 (5.23)	29.83 (3.72)	28.42 (3.21)
BS	7.20 (0.63)	6.03–8.59	7.16 (0.42)	7.04 (0.62)	7.06 (0.98)	7.31 (0.61)	7.03 (0.57)

ICV: intracranial volume; TTV: total tissue volume; cGM: cortical gray matter; WM: white matter; CSF: cerebrospinal fluid; dGM: deep gray matter; HPC: hippocampus; AM: amygdala; CB: cerebellum; BS: brainstem; EP: extremely preterm; VP: very preterm; MP: moderately preterm; LP: late preterm; FT: full term.

## Data Availability

The data presented in this study are available on request from the corresponding author due to missing consent from participants to share data online.
